# Boldine Ameliorates Estrogen Deficiency-Induced Bone Loss via Inhibiting Bone Resorption

**DOI:** 10.3389/fphar.2018.01046

**Published:** 2018-09-13

**Authors:** Kun Chen, Zheng-tao Lv, Peng Cheng, Wen-tao Zhu, Shuang Liang, Qing Yang, Virginia-Jeni Akila Parkman, Chen-he Zhou, Xing-zhi Jing, Hui Liu, Yu-ting Wang, Hui Lin, Hui Liao, An-min Chen

**Affiliations:** ^1^Department of Orthopedics, Tongji Hospital, Tongji Medical College, Huazhong University of Science and Technology, Wuhan, China; ^2^Biological Engineering and Regenerative Medicine Center, Tongji Hospital, Tongji Medical College, Huazhong University of Science and Technology, Wuhan, China; ^3^Department of Oral Medicine, Infection, and Immunity, Harvard School of Dental Medicine, Boston, MA, United States; ^4^Department of Orthopaedic Surgery, The Second Affiliated Hospital, School of Medicine, Zhejiang University, Hangzhou, China; ^5^Department of Orthopaedic Surgery, Wuhan Union Hospital, Tongji Medical College, Huazhong University of Science and Technology, Wuhan, China

**Keywords:** boldine, osteoporosis, osteoclast, osteoblast, AKT

## Abstract

Osteoporosis is an enormous health problem caused by the imbalance between bone resorption and bone formation. The current therapeutic strategies for osteoporosis still have some limitations. Boldine, an alkaloid isolated from *Peumus boldus*, has been shown to have antioxidant and anti-inflammatory effects *in vivo*. For the first time, we discover that boldine has a protective effect for the estrogen deficiency-induced bone loss in mice. According to the Micro-CT and histomorphometry assays, boldine conducts this protective effect through inhibiting bone resorption without affecting bone formation *in vivo*. Moreover, we showed that boldine can inhibit receptor activator of nuclear factor-κB ligand (RANKL)-induced osteoclast formation via impairing the AKT signaling pathways, while SC79 (an AKT agonist) partially rescue this effect. In conclusion, our results suggest that boldine can prevent estrogen deficiency-induced osteoporosis by inhibiting osteoclastogenesis. Thus, boldine may be served as a novel therapeutic agent for anti-osteoporotic therapy.

## Introduction

Osteoporosis is one of several major health problems, affecting 10 million people in the United States and 27.6 million in the Europe (Zhou et al., 2018). It is induced by the imbalance between bone formation and bone resorption NIH Consensus Development Panel on Osteoporosis Prevention, Diagnosis, and Therapy (2001). This imbalance results in decreased bone mass and abnormal bone architecture, increasing the risk of fragility fracture (Canalis et al., 2007). Post-menopausal osteoporosis is one of the major types of osteoporosis stems from the cessation of ovarian function at menopause and from genetic and non-genetic factors which heighten and prolong the rapid phase of bone loss characteristic of the early post-menopausal period (D’Amelio et al., 2008). By 2025, the prevalence of osteoporosis in the United States is estimated to be more than 14 million, incurring $25.3 billion in costs (Johnell and Kanis, 2006). Therefore, the preventions and treatments of this disease are of significant importance.

The current therapeutic strategies for post-menopausal osteoporosis mainly focus on improving bone mineral density (BMD) and reducing the risk of fragility fracture (Khosla and Hofbauer, 2017). Pharmacological agents against osteoporosis include anti-resorptive drugs which decrease bone resorption to produce secondary gains in bone mass, and anabolic drugs, which directly stimulate increases in bone mass (Bernabei et al., 2014). A number of drugs have been developed and used for prevention and treatment of osteoporosis during the last 40 years, including estrogen, calcitonin, selective estrogen-receptor modulator (SERM; raloxifene), bisphosphonates, and monoclonal antibodies against the receptor activator of nuclear factor-κB ligand (RANKL; denosumab), parathyroid hormone analog teriparatide, and parathyroid hormone-related peptide analog (abaloparatide) (Neer et al., 2001; Riggs and Hartmann, 2003; Kearns et al., 2008; Khosla et al., 2012; Miller et al., 2016; Shirley, 2017). Despite these dramatic advances, there are still some defects that remain. For example, bisphosphonates, the most extensively used anti-resorptive drugs for osteoporosis, may cause side-effects such as osteonecrosis of the jaw and atypical fractures, as well as unproven efficacy after 5 years of treatment (Khosla et al., 2007; Whitaker et al., 2012; Shane et al., 2014). Teriparatide, a type of anabolic drug, is limited for up to 24 months treatment duration by the United States Food and Drug Administration (FDA) because of an increasing risk of osteosarcoma in growing rodents treated with high doses (Vahle et al., 2004). These side-effects and uncertain long-term efficacy is leading many patients, who could have benefit from the drugs treatment, to not take these drugs (Khosla and Hofbauer, 2017). Recently, there are increasing reports about the involvement of immune cells, including T cells and B cells, on the pathogenesis of post-menopausal osteoporosis (D’Amelio et al., 2008; Mori et al., 2015). These findings lead to the identification of new therapeutic targets and biological drugs for treating post-menopausal osteoporosis. However, there is still an urgent clinical need to find drugs which could treat osteoporosis efficiently without any severe side-effects.

Boldine([s]-2,9-dihydroxy-1,10-dimethoxyaporphine), the major alkaloid found in the leaves and bark of *Peumus boldus*, has been reported to have antioxidant, hepatoprotective, cytoprotective, and anti-inflammatory effects (Kringstein and Cederbaum, 1995; Bannach et al., 1996; Jang et al., 2000; Lau et al., 2015). Although there is no report about the effects of boldine on osteoporosis now, it has been shown recently that boldine can inhibit osteoclast formation in collagen-induced arthritis (Zhao et al., 2017). Moreover, boldine has shown some effects on inhibiting the NF-κB, extracellular signal-regulated kinase (ERK) and AKT signaling, which are important signaling pathways involved in osteoclast differentiation and function (Gerhardt et al., 2014; Pandurangan et al., 2016; Yang et al., 2018). Taken all together, there is a high possibility that boldine can affect bone remodeling and osteoporosis. Here, we aim to explore the effects of boldine on ovariectomy (OVX)-induced osteoporosis and to demonstrate the underlying mechanisms involved.

## Results

### Boldine Ameliorated Estrogen-Deficiency Induced Bone Loss in Mice

**FIGURE 1:**
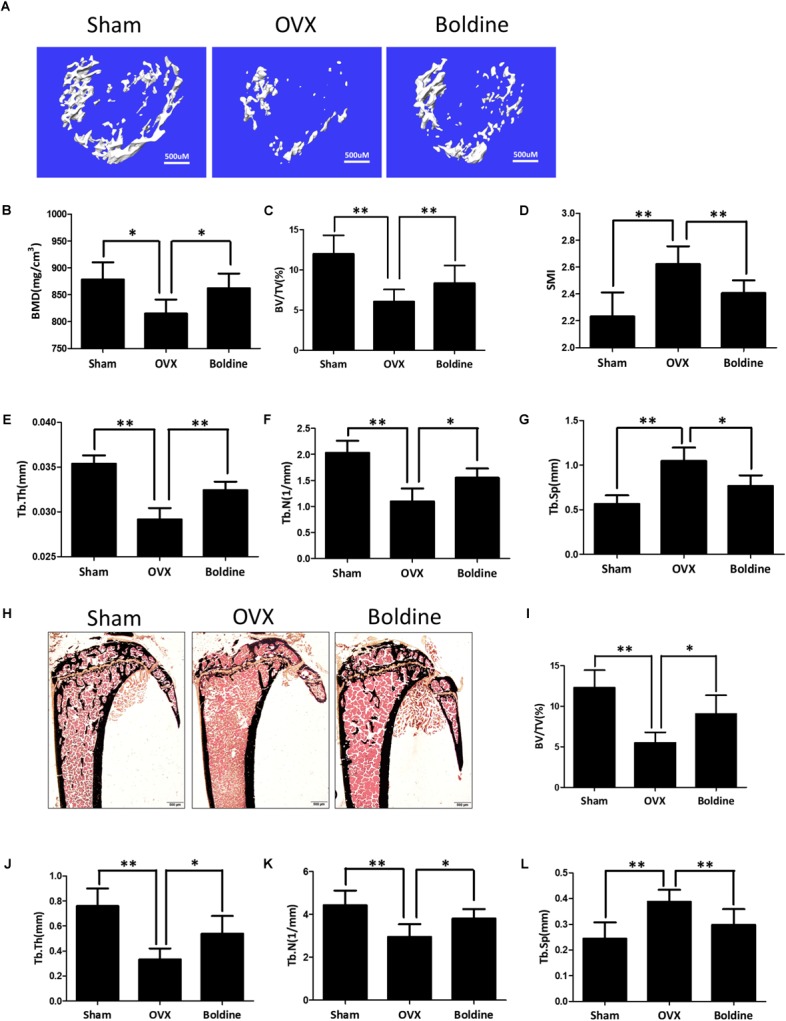
Boldine inhibits OVX-induced bone loss in mice. 8-week-old female wild type mice were randomly divided into three groups: Sham (Sham operation + Vehicle treatment), OVX (OVX operation + Vehicle treatment), and Boldine (OVX operation + Boldine treatment). After the operation, mice were treated with either vehicle or boldine (20 mg/kg/d) for 6 weeks. **(A)** Representative von Kossa staining images of tibias from each group. Scale bar = 500 μm. **(B–E)** Histomorphological sections were stained, the BV/TV, Tb.Th, Tb.N, and Tb.Sp were measured and quantified. **(F)** Representative 3D micro-CT images of trabecular bone from each group. **(G–L)** The BMD, BV/TV, SMI, Tb.Th, Tb.N, and Tb.Sp values from each group were obtained and analyzed with the built-in software of Micro-CT. All data: mean ± SD, *n* = 8. ^∗^*p* < 0.05 and ^∗∗^*p* < 0.01.

To explore the effect of boldine on osteoporosis, we used a murine model of OVX-induced osteoporosis. 8-week-old female wide type mice were randomly divided into three groups: Sham (Sham + Vehicle), OVX (OVX + Vehicle), and Boldine (OVX + Boldine). After the OVX or sham surgery, mice were treated with vehicle or boldine (20 mg/kg/d) for 6 weeks. Then the mice were sacrificed, the left tibias from each mouse were scanned for micro-CT analysis. As expected, OVX group showed dramatic bone loss compared to the Sham group, as revealed by decreased BMD, trabecular bone BV/TV, trabecular thickness (Tb.Th), trabecular number (Tb.N), increased structural model index (SMI) and trabecular separation (Tb.Sp) (**Figures 1F–L**). Meanwhile, the boldine treatment group showed a protective effect when compared to OVX group, as shown by elevated BMD, BV/TV, Tb.Th, Tb.N, and decreased SMI and Tb.Sp (**Figures 1A–G**). These data suggest that boldine treatment ameliorated OVX-induced bone loss in mice.

To further confirm that, the right tibia of each mouse was dissected for histomorphometry assays. As expected, the von Kossa staining and histomorphological quantification data showed a significant drop in bone volume in OVX group mice in comparison to the Sham group, suggesting that this osteoporosis model was set up successfully (**Figures 1H,I**). Meanwhile, the bone volume/total volume (BV/TV) in the boldine treatment group was significantly higher than OVX group (**Figures 1H,I**). Moreover, the histomorphological quantification demonstrated a significant elevation of Tb.N, and Tb.Th in the boldine treatment group when compared to the OVX group, and a significant drop in Tb.Sp (**Figures 1J–L**). All of these data are in accordance with the micro-CT assays, suggesting that boldine treatment can partially rescue OVX-induced bone loss in mice.

### Boldine Inhibited OVX Induced Bone Resorption Without Affecting Bone Formation *in vivo*

**FIGURE 2:**
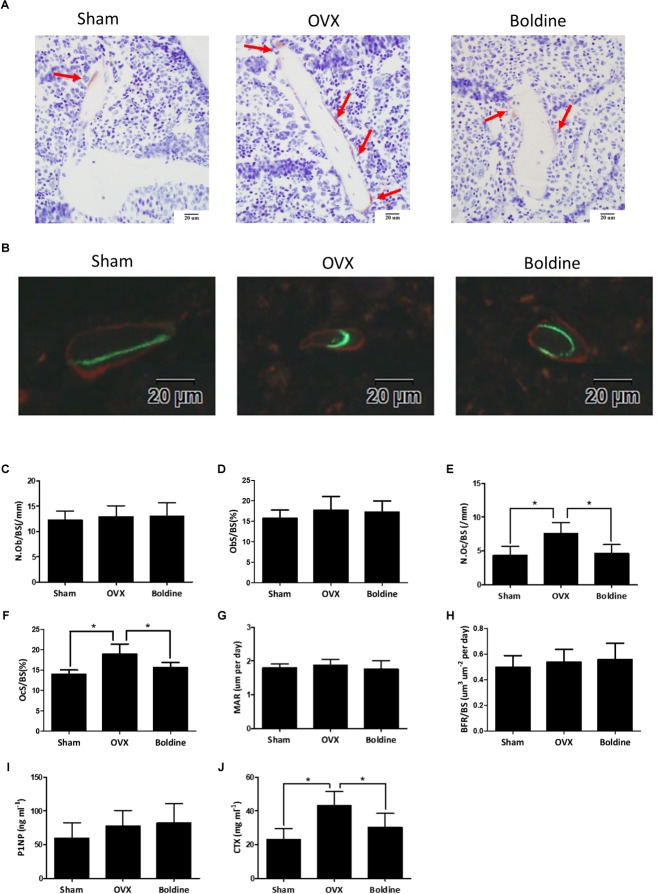
Boldine inhibits OVX-induced bone resorption without affecting bone formation in mice. Mice were randomly divided into three groups: Sham (Sham + Vehicle), OVX (OVX + Vehicle), and Boldine (OVX + Boldine), and were sacrificed after treatment with vehicle or boldine (20 mg/kg/d) for 6 weeks. Calcein and alizarin red were injected 9 and 2 days separately prior to sacrifice. **(A)** Representative TRAP staining images of the tibia plastic sections from each group. Red arrows indicate stained multinuclear osteoclasts. Scale bar = 20 μm. **(B)** Representative images of calcein (green) and alizarin red (red) labels for bone formation measurement. Scale bar = 20 μm. **(C–H)** The N.Ob/BS, ObS/BS, N.Oc/BS, OcS/BS, MAR, and BFR/BS were measured and quantified from plastic sections. **(I,J)** The serum level of P1NP and CTX were measured. All data: mean ± SD, *n* = 8. ^∗^*p* < 0.05 and ^∗∗^*p* < 0.01.

Since osteoporosis is induced by the imbalance between bone formation and bone resorption, the current therapeutic strategies mainly focus on increasing bone formation and/or decreasing bone resorption (Tabatabaei-Malazy et al., 2017). In order to better understand the mechanism by which boldine inhibits OVX induced bone loss, we next examined the bone formation and bone resorption in OVX mice treated with boldine. According to our data, OVX mice exhibited increased bone resorption without significant changes in bone formation when compared to sham mice, as shown by increased number of osteoclasts normalized to the bone surface (N.Oc/BS), increased surface area of osteoclast to bone surface area (OcS/BS), no significant difference in number of osteoblasts normalized to the bone surface (N.Ob/BS), no changes in surface area of osteoblast to bone surface area (ObS/BS), mineral apposition rate (MAR) and bone formation rate to bone surface (BFR/BS) (**Figures 2A–H**). When we compared boldine treatment mice with OVX mice, boldine treatment partially inhibited OVX-induced bone resorption without affecting bone formation, as revealed by significant decreases in N.Oc/BS and OcS/BS, as well as there being no significant difference in N.Ob/BS, ObS/BS, MAR, and BFR/BS (**Figures 2A–H**). In addition, we measured the serum bone formation marker procollagen 1 N-terminal peptide (P1NP), and serum bone resorption marker type I collagen cross-linked C-terminal telopeptide (CTX-1) (Rosen et al., 2000; Thurairaja et al., 2006). As expected, the OVX group exhibited a higher CTX-1 level and no difference in P1NP level when compared to the Sham group (**Figures 2I,J**). Moreover, the boldine treatment group showed a significant decrease in CTX-1 level and no difference in P1NP when compared to the OVX group (**Figures 2I,J**), indicating a lower bone resorption ability without changes in bone formation. Taken together, these data suggest that boldine treatment ameliorates OVX-induced osteoporosis through inhibiting bone resorption without affecting bone formation *in vivo*.

### Boldine Inhibited Osteoclast Formation and Function *in vitro*

**FIGURE 3:**
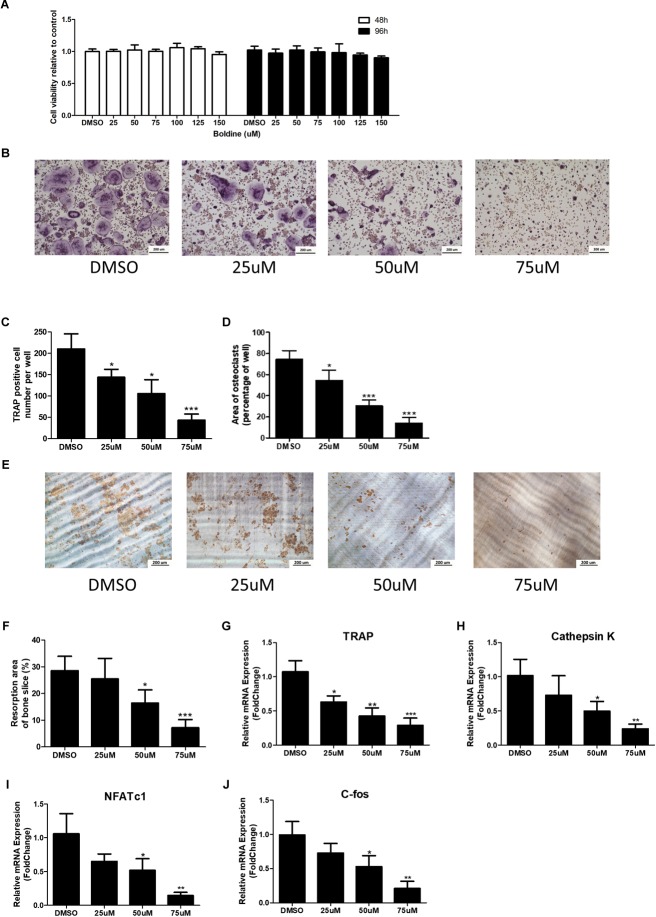
Boldine inhibits osteoclast formation and function *in vitro*. **(A)** BMMs were isolated from wide type mice and seeded into 96 wells at the same density (2.5^∗^10^3^ cell/cm^2^). Then cells were treated with DMSO or different concentrations of boldine in the presence of M-CSF for 48 or 96 h. The CCK-8 kit was used to quantify the cell viability. **(B)** Representative TRAP staining images from BMMs treated with DMSO or different doses of boldine in the presence of M-CSF and RANKL for 4 days. Scale bar = 200 μm. **(C,D)** Number and area of TRAP positive multinuclear osteoclasts (≥3 nuclei) from each 96 well were measured and quantified. **(E,F)** BMMs were cultured in osteoclastogenic medium for 4 days, then reseeded on dentin slice treated with vehicle or different doses of boldine for another 2 days. **(E)** Representative pictures of bone resorption pits and **(F)** quantified bone resorption area. **(G–J)** mRNA expression of *Trap, Cathepsin K, NFATc1*, and *C-fos* of BMMs treated with vehicle or indicated doses of boldine in the presence of M-CSF and RANKL for 4 days. All data: mean ± SD, *n* = 3. ^∗^*p* < 0.05, ^∗∗^*p* < 0.01, and ^∗∗∗^*p* < 0.001.

To examine if boldine has some effect on osteoclastogenesis, we used bone marrow-derived macrophages (BMMs) and induced them to differentiate into osteoclasts *in vitro*. First, we did the cytotoxicity assays for the BMMs, which were treated with vehicle (DMSO) or different doses of boldine (25, 50, 75, 100, 125, and 150 μM) in the presence of 30 ng/mL macrophage colony-stimulating factor (M-CSF) for 24 or 48 h. No toxicity to boldine was detected even at a relative high dose of 150 μM (**Figure 3A**). We then stimulated BMMs with 30 ng/ml M-CSF and 10 ng/ml RANKL to induce them to differentiate into osteoclasts. Tartrate-resistant acid phosphatase (TRAP) staining results suggested that boldine inhibited osteoclastogenesis in a dose depend manner (**Figure 3B**). When we treated BMMs with 25 μM of boldine, TRAP positive multinucleated cell number decreased significantly compared to vehicle group, and there were only 25% TRAP positive cells left when treated BMMs with 75 μM of boldine (**Figure 3C**). The total area of osteoclasts also decreased at the same time (**Figure 3D**). Consistently, gene expression analysis revealed that the expression of the osteoclast specific genes including TRAP, Cathepsin K, C-fos, and nuclear factor of activated T cells c1 (NFATc1), the master transcriptional factor during osteoclast differentiation, decreased in boldine treatment groups in a dose dependent manner (**Figures 3G–J**).

Furthermore, pit formation assay was performed to test if boldine can affect osteoclast bone resorption function (Ghayor et al., 2011). BMMs were seeded on collagen I coated plates in the presence of 30 ng/ml M-CSF and 10 ng/ml RANKL for 4 days to induce mature osteoclasts. These osteoclasts were then digested and reseeded on dentin slices in 96 wells in the same density (1000 cells/well), continued treating with 30 ng/ml M-CSF and 10 ng/ml RANKL together with vehicle or boldine (50 μM) for another 2 days. After that, bone slices were collected, and osteoclast resorption pits were stained. Our data showed that boldine decreased bone resorption pit area in a dose dependant manner, indicating that boldine prohibits osteoclast bone resorption function (**Figures 3E,F**). All of this data suggested that boldine inhibited osteoclast formation and osteoclast bone resorption function *in vivo*.

### Boldine Had No Effects on Osteoblast Formation *in vitro*

**FIGURE 4:**
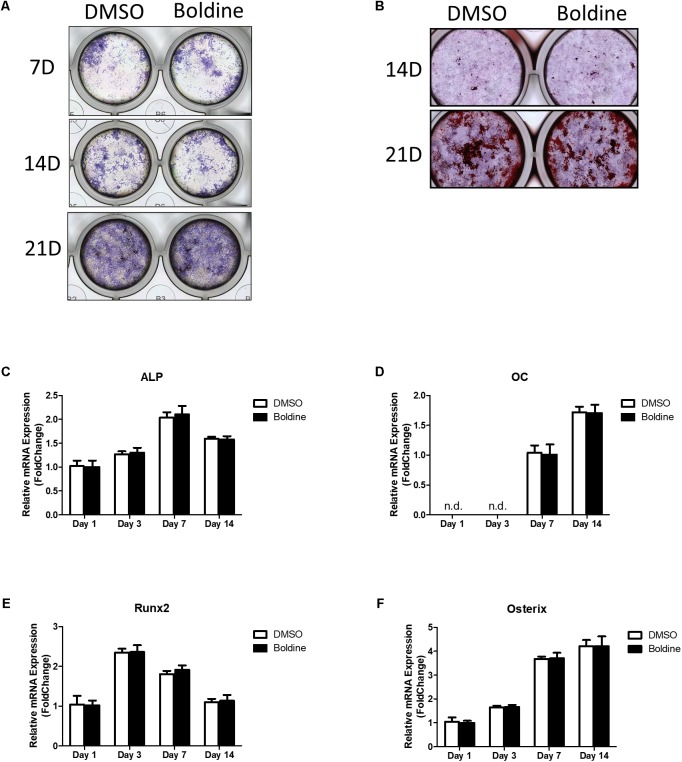
Boldine has no effect on osteoblast formation *in vitro*. BMSCs were isolated from wild type mice, cultured in osteogenic medium treated with vehicle or boldine (50 μM) for an indicated time. **(A,B)** Representative ALP staining **(A)** and Alizarin red **(B)** staining pictures. **(C–F)** mRNA expression of *ALP, OC, Runx2*, and *Osterix*.

Our *in vivo* data suggested that boldine treatment didn’t affect bone formation in mice (**Figures 2G–I**). To further confirm that, we next examined the effect of boldine on osteoblast bone formation *in vitro*. Primary bone marrow stem cells (BMSCs) were isolated from male 4-week-old wide type C57BL/6 mice, cultured in osteogenic medium for 1, 3, 7, 14, or 21 days with treatment of vehicle or boldine. Alkaline phosphatase (ALP) staining and alizarin red staining suggested no difference between vehicle and boldine treatment groups (**Figures 4A,B**). Consistently, the gene expression of osteoblastic markers RUNX2, osteocalcin (OC), ALP, and Osterix showed no significant difference between groups with or without treatment of boldine (**Figures 4C–F**). These data demonstrate that boldine have no effects on osteoblast formation *in vitro*, which is in accordance with our *in vivo* data.

### Boldine Inhibited RANKL-Induced Activation of the AKT Pathway During Osteoclastogenesis

**FIGURE 5:**
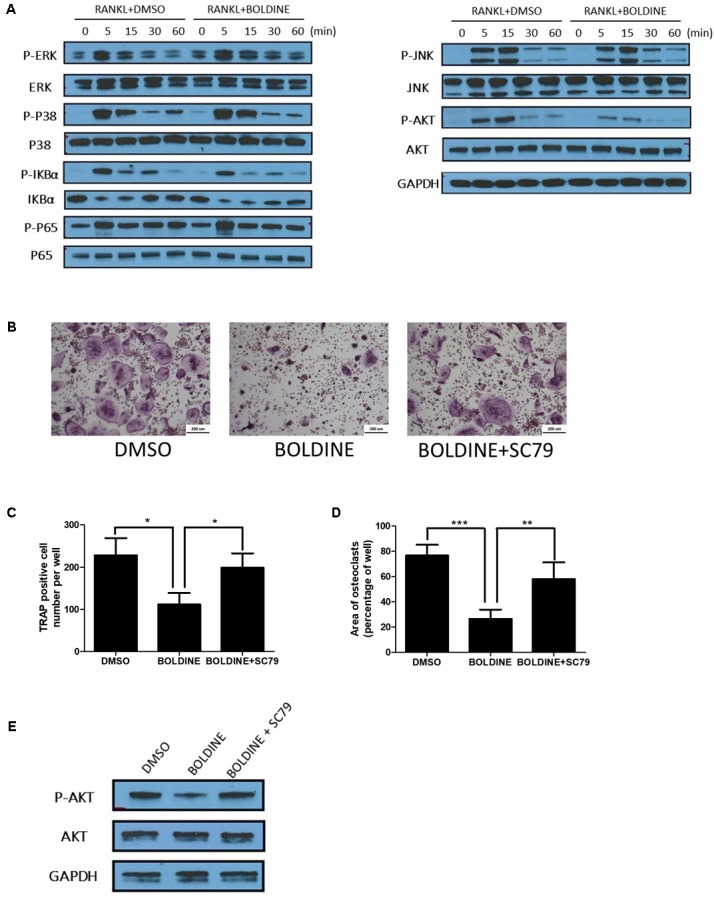
Boldine inhibited RANKL-induced activation of AKT pathways *in vitro*. **(A)** BMMs were isolated from wild type mice, seeded into 6 wells at the same density (2.5^∗^10^3^ cell/cm^2^), treated with M-CSF for 1 day, pre-treated with DMSO or boldine (50 μM) for 2 h, then added RANKL for 0, 5, 15, 30, and 60 min. Total proteins from the cells were collected and WB was performed. **(B–D)** BMMs were isolated, treated with vehicle or boldine or boldine + SC79 for 4 days in the presence of M-CSF and RANKL. **(B)** Representative pictures of TRAP staining. Numbers **(C)** and area **(D)** of TRAP positive multinuclear osteoclasts (≥3 nuclei). **(E)** BMMs were pre-treated with vehicle or boldine (50 μM) for 2 h, then stimulated with RANKL in the presence of vehicle or SC79 for 30 min. Cells were collected and WB for P-AKT, AKT and GAPDH was performed. All data: mean ± SD, *n* = 3. ^∗^*p* < 0.05, ^∗∗^*p* < 0.01, and ^∗∗∗^*p* < 0.001.

Given our data that boldine inhibited bone resorption *in vivo* and *in vitro*, we next explored the underlying mechanism by which boldine affected osteoclasts. It has been reported that AKT, NF-κB and mitogen-activated protein kinases (MAPK) signaling pathways are critical pathways involved in osteoclast differentiation and activation (Lee et al., 2001; Li et al., 2002; Clohisy et al., 2004). There are several papers in which it was reported that boldine affects these signaling pathways (Gerhardt et al., 2014; Pandurangan et al., 2016; Yang et al., 2018). As a result of these reports, we performed western blot (WB) to explore the effects of boldine on these signaling pathways during osteoclastogenesis. As expected, upon treatment, BMMs showed the activation of RANKL, AKT, NF-κB, and MAPK signaling pathways, as revealed by the increased phosphorylation levels of AKT, c-Jun N-terminal kinase (JNK), ERK1/2, p38, factor of kappa light polypeptide gene enhancer in B-cells inhibitor alpha (IkBa), and P65 (**Figure 5A**). Moreover, the treatment of boldine ameliorated RANKL induced phosphorylation of AKT, without affecting NF-κB and MAPK signaling pathways (**Figure 5A**). These findings suggested that boldine might inhibit osteoclast formation via inhibiting RANKL-induced activation of the AKT pathway during osteoclastogenesis.

### The AKT Agonist Partly Reversed the Inhibitory Effect of Boldine on Osteoclastogenesis

As shown before, our data suggested that boldine inhibited RANKL-induced activation of AKT pathway. We next combined boldine with SC79, an AKT agonist, to test if the activation of AKT signaling could reverse boldine’s inhibitory effect on osteoclastogenesis. WB results showed that SC79 increased p-AKT levels in boldine treated BMMs (**Figure 5E**). TRAP staining results suggested that, when compared to the boldine treated group, the boldine and SC79 co-treated group had more TRAP positive multinucleated cells (**Figure 5B**). Moreover, the quantification showed significantly increased TRAP positive multinucleated cell number and elevated TRAP positive area (**Figures 5C,D**), indicating a stronger osteoclast formation ability in boldine and SC79 co-treated group when compared to the boldine treated group. Taken together, this data indicated that AKT agonist could partially reverse the suppressive effect of boldine on osteoclastogenesis, suggesting that boldine could inhibit osteoclastogenesis through inhibiting AKT signaling.

## Discussion

Osteoporosis, a bone metabolism disease which is characterized by decreased BMD and increased risk of fragility fractures, is considered to be one of the major public health problems (Willson et al., 2015). Post-menopausal osteoporosis is one of the major types of osteoporosis which is the most prevalent disease in menopausal women, and is strongly associated with low quality of life (Ji and Yu, 2015). Post-menopausal osteoporotic fractures result in substantial morbidity and mortality, placing a heavy economic burden on individuals (Cauley, 2017). A number of drugs have been discovered to treat osteoporosis, including estrogen, calcitonin, SERM, bisphosphonates and so on (Pavone et al., 2017). There are also increasing reports about the involvement of immune cells on the pathogenesis of post-menopausal osteoporosis (Mori et al., 2015). For example, D’Amelio et al. (2008) have demonstrated a fundamental role for T cells in post-menopausal bone loss. These findings provide a novel explanation for the propensity to osteopenia and osteoporosis development after the cessation of ovarian function and give us a hint to find new biological drugs for treating post-menopausal osteoporosis. The drugs current used for treating osteoporosis are efficient, but far from ideal, making an urgent need to find new pharmacological agents that can treat osteoporosis efficiently without serious side-effects.

Mounting evidence has shown that alkaloids extracted from herbs have promise in protecting against bone loss (Lee et al., 2010; Takahashi et al., 2012; Wang et al., 2018). Boldine, an isoquinoline alkaloid extracted from the leaves and bark of *P. boldus*, has been reported to have antioxidant, hepatoprotective, cytoprotective, and anti-inflammatory effects (Kringstein and Cederbaum, 1995; Bannach et al., 1996; Jang et al., 2000; Lau et al., 2015). However, there is no report about the effects of boldine on osteoporosis. In the present study, our results show that boldine treatment (20 mg/kg/d) could significantly increase BV/TV and BMD in OVX mice, suggesting that boldine could ameliorate estrogen deficiency-induced osteoporosis.

Current pharmacological agents against osteoporosis mainly focus on decreasing bone resorption (anti-resorptive drugs), or increasing bone formation (anabolic drugs) (Bernabei et al., 2014). The major problem for current anti-resorptive drugs is that the bone resorption inhibition effect is always accompanied by the inhibited bone formation because of the coupling of these two processes (Martin and Sims, 2005). For example, bisphosphonates, the most widely used anti-resorption drug for osteoporosis, could cause osteonecrosis of jaw and atypical fractures (Khosla et al., 2007; Whitaker et al., 2012; Shane et al., 2014). Hence, agents that inhibit bone resorption without affecting bone formation are more desirable. We also performed histomorphometry analysis to examine the effect of boldine on bone metabolism. Our data showed that boldine treatment induced significant decreases in N.Oc/BS and OcS/BS in OVX mice, suggesting that boldine has an inhibitory effect on bone resorption. Meanwhile, no significant difference was found in BFR, MAR, OcS/BS, and N.Ob/Bs, indicating that boldine doesn’t affect normal bone formation. The decreased CTX value and unchanged P1NP value in the boldine treatment group when compared to the OVX group further confirms that. Moreover, our *in vitro* data showed that boldine inhibited osteoclastogenesis in a dose dependent manner, together with the decreased expression of osteoclast specific genes including TRAP, Cathepsin K, DC-STAMP, and NFATc1. On the other hand, ALP staining, alizarin red staining and real-time PCR (RT-PCR) results suggest that boldine treatment doesn’t affect osteoblast formation and osteoblast gene expression during osteogenic differentiation. In all, our data suggest that boldine can inhibit bone resorption without affecting bone formation both *in vivo* and *in vitro*, making it a promising drug for osteoporosis.

Osteoclast differentiation is a complex process regulated by multiple signaling cascades. Among these various signaling pathways, AKT, which is activated by both RANKL and M-CSF, is a central player in the regulation of osteoclast differentiation and survival (Gingery et al., 2003; Moon et al., 2012). It has been reported that boldine can inhibit the activation of AKT in T24 human bladder cancer cell line (Gerhardt et al., 2014). We used WB to examine the effect of boldine on AKT signaling during osteoclastogenesis. As expected, when treated with RANKL, the phosphorylation level of AKT increased and reached highest at 15 min. Moreover, boldine inhibited the phosphorylation level of AKT significantly. These data hinted that boldine inhibited osteoclastogenesis via inhibiting the activation of AKT. To further confirm that, AKT agonist SC79 was used to treat BMMs together with boldine during osteoclastogenesis. The TRAP staining and pit assay data suggested that SC79 can partially reserve the inhibitory effect of boldine. It has also been reported that boldine can inhibit JNK, MAPK, and NF-κB signaling pathways (Gerhardt et al., 2014; Pandurangan et al., 2016; Yang et al., 2018). Considering that these signaling pathways are all involved during osteoclast differentiation, we tested them as well. However, no significant differences were found between vehicle and boldine treatment groups. It is possible that the concentration of boldine we used (50 μM), which could inhibit osteoclastogenesis efficiently, is still too low to influence these signaling pathways.

In conclusion, our data suggested that boldine ameliorated estrogen deficiency-induced bone loss via inhibiting bone resorption without affecting bone formation *in vivo*. Moreover, we showed that boldine exerts its inhibitory effects on osteoclast formation and bone resorption via impairing the activation of the AKT signaling pathway. Therefore, we concluded that boldine might be a novel therapeutic agent for preventing osteoporosis.

## Materials and Methods

### Medium and Reagents

Boldine was purchased from Sigma-Aldrich (St. Louis, MO, United States), SC79 was purchased from Selleck Chemicals (Houston, TX, United States). These reagents were dissolved in DMSO and stored at -20°C until being used in experiments. Recombinant murine soluble RANKL and macrophage colony-stimulating factor (M-CSF) were purchased from R&D systems (Minneapolis, MN, United States), dissolved in 0.1% BSA and stored at -80°C before being used. Minimum essential medium eagle–alpha modification (α-MEM) medium, penicillin/streptomycin and FBS were gained from Gibco-BRL (Sydney, Australia). Cell counting kit–8 (CCK-8) and acid phosphatase kit for TRAP kit were gained from Sigma-Aldrich (St. Louis, MO, United States). The Prime Script RT reagent kit and SYBR used for RT-PCR experiments were gained from TaKaRa Biotechnology (Otsu, Shiga, Japan). Primary antibodies of ERK (#4695), P38 (#9212), IKBα (#4818), P65 (#8242), AKT (#4691), JNK (#9252), P-ERK (Thr^202^/Tyr^204^) (#4370), P-P38 (Thr^180^/Tyr^192^) (#4511), P-P65 (Ser^536^) (#3033), P-IKBα (Ser^32^) (#2859), P-AKT (Ser^473^) (#4051), and P-JNK (Thr^183^/Tyr^185^) (#4668) were purchased from Cell Signaling Technology (Cambridge, MA, United States). Primary antibody of GAPDH was gained from BOSTER (BM3876, BOSTER, Wuhan, China). HRP conjugate secondary antibodies including anti-rabbit IgG and anti-mouse IgG were gained from Promega (Fitchburg, WI, United States). ELISA kits used to test serum level of CTX-1 and P1NP were obtained from Immunodiagnostic Systems Limited (The Boldons, United Kingdom).

### Establishment of Marine Ovariectomized (OVX)-Induced Osteoporosis Model

The animal experiment was designed and carried out in accordance with the Guide for the Care and Use of Laboratory Animals promulgated by the United States National Institutes of Health (NIH), and was approved by the Ethics Committee on Animal Experimentation of Tongji Medical College, Huazhong University of Science and Technology (Wuhan, China). Thirty 8-week-old wild type C57/BL6 mice were purchased from the Experimental Animal Center of Tongji Medical College (Wuhan, China) and were divided randomly into three groups: Sham (Sham operation + vehicle treatment), OVX (OVX operation + vehicle treatment); Boldine (OVX operation + Boldine treatment). Mice were injected intramuscular xylazine (2 mg/kg) and ketamine (50 mg/kg) to anesthetize. Then sham or OVX operation was done as described previously (Zhou et al., 2018). For the boldine treatment group, boldine powder was first dissolved in 100% ethanol to make a stock solution (25 mg/ml) and stored at -20°C. When using, the stock solution of boldine was dissolve in distill water 1:4 (v/v) to make a working solution (5 mg/ml) and was given to mice by oral gavage 4 ml/kg 5 days a week. For the mice in Sham and OVX group, vehicle (20% ethanol in distilled water, 4 ml/kg) was given by oral gavage 5 days a week. After a total period of 6 weeks treatment, all mice were sacrificed with an overdose injection of pentobarbital (90 mg/kg). Calcein (20 mg/kg) and alizarin red (30 mg/kg) were injected 9 and 2 days, respectively, before sacrifice. All tibias were dissected and fixed in 70% ethanol, left tibias were sent for Micro-CT analysis and right tibias were sent for histomorphometry assays. The blood was also collected, the serum was isolated and stored at -80°C until used for ELISA analysis.

### Micro-Computed Tomography (μ-CT)

After removing the soft tissues, the proximal tibias were analyzed by microcomputed tomography (μ-CT) system (μ-CT50 Scanco Medical, Bassersdorf, Switzerland) using a source voltage of 80 kV and 80 mA source current with a voxel size of 10 mm. The region at a distance of 0.5 mm below the growth plate was set as the region of interest (ROI) for analysis. All the data including BMD, BV/TV, SMI, Tb.N, Tb.Th, and Tb.Sp were analyzed with the built-in software of the μ-CT. The three-dimensional bone structure image slices were reconstructed using Avizo 9.1 software (ThermoFisher Scientific, Oregon, United States).

### Histomorphological Analysis

All the right tibias were dissected, fixed in 70% ethanol for 3 days. After fixation, tibias were infiltrated with a mixture of 85% methyl methacrylate (Sigma-Aldrich), 15% dibutyl phthalate (Sigma-Aldrich), and 0.15% benzoyl peroxide (PolyScience) at 4°C for 9 days. Tibias were then embedded in a mixture of 85% methyl methacrylate, 15% dibutyl phthalate, and 3% benzoyl peroxide, polymerized at 37°C. Standard undecalcified sections (4 μm) were cut along the coronal plane from anterior to posterior using a Reichert-Jung microtome (Cambridge Scientific). Subsequently, von Kossa staining, Toluidine Blue and TRAP staining were performed, quantitative bone histomorphometric measurements were analyzed using the OsteoMeasure system (OsteoMetrics). The representative pictures of TRAP staining and labeling from the plastic sections were taken using Nikon Eclipse E800 microscopy (Nikon) at 400× magnification.

### Bone Turnover Analysis

The blood was collected using blood collection tubes (#365967 BD Microtainer SST, Becton Dickinson, Franklin Lakes, NJ, United States), and centrifuged at 2,000 rpm for 25 min to isolate serum. Then the serum levels of CTX-1 and P1NP were measured using the ELISA kit (IDS Nordic, Herlev, Denmark).

### BMMs Isolation, Osteoclast Differentiation and TRAP Staining *in vitro*

Primary bone marrow cells were flushed from the long bones of male 6-week-old wide type C57BL/6 mice and then plated on non-treated plates in α-MEM containing 15% FBS, 1% penicillin and 10 ng/ml M-CSF for 3 days. Subsequently, cells were washed with PBS and harvested with 0.05 mM EDTA in PBS for 20 min. Then BMMs were counted, plated 2.5^∗^10^3^ cell/cm^2^ in 6, 12, or 96 wells for further experiments.

To induce osteoclast differentiation, the isolated BMMs were cultured in osteoclastogenic medium (α-MEM containing 15% FBS, 1% penicillin, 30 ng/ml M-CSF and 10 ng/ml RANKL) for 4 days in the presence of indicated doses of boldine with or without 8 μg/ml SC79. Then, the cells were fixed with 4% PFA for 15 min and performed TRAP staining using the TRAP staining kit (387A-1KT, Sigma-Aldrich). Multinuclear (≥3 nuclei) TRAP positive cells were recognized as osteoclasts and the staining picture were taken using BZ-X710 microscope (Keyence, Osaka, Japan). The osteoclasts number and area were measured using ImageJ software.

### Cell Viability Assay

The isolated BMMs were seeded into 96 wells in the same density (2.5^∗^10^3^ cell/cm^2^). Then the cells were treated with DMSO or different concentrations of boldine in the presence of M-CSF (30 ng/ml) in α-MEM containing 15% FBS, 1% penicillin for 48 or 96 h. The CCK-8 kit (96992, Sigma-Aldrich) was used to quantify the cell viability according to the kit manual.

### Bone Resorption Assay

The primary BMMs were isolated, seeded on collagen-coated plates in the density of 2.5^∗^10^3^ cell/cm^2^ and cultured in osteoclastogenic medium for 4 days until mature osteoclasts were formed. Then mature osteoclasts were digested and seeded on dentin slice in 96 wells (1000 cells/well). Subsequently, the osteoclasts were continued cultured in osteoclastogenic medium in the presence of indicated dosages of boldine. After 48 h, the dentin slices were obtained and wiped with q-tips to remove cells, pit lection staining was performed. The brown area on the dentin slice was recognized as the bone resorption area during the 48 h. The representative pictures were taken using Nikon Eclipse E800 microscopy (Nikon), and the resorption area was measured using ImageJ software.

### BMSCs Isolation, Osteoblast Differentiation

Primary bone marrow cells were flushed from the long bones of male 4-week-old wide type C57BL/6 mice and then plated on treated plates in basic medium (α-MEM containing 10% FBS and 1% penicillin) for 8 days. Then BMSCs were digested and seeded on 24 wells in the same density (10,000 cells/well), cultured until confluent.

When the cell confluency is achieved, the basic medium was changed to osteogenic medium (α-MEM containing 10% FBS, 1% penicillin, 50 μg/ml Ascorbic acid, and 10 mM β-glycerol phosphate) to induce osteoblast differentiation with or without treatment of boldine (50 μM). At 7, 14 or 21 days, the differentiated cells were fixed, ALP and Alizarin Red staining were performed.

### RNA Isolation and Quantitative RT-PCR

Quantitative RT-PCR was performed as described previously (Zhou et al., 2018). In brief, BMMs or BMSCs were plated in 24 wells and cultured with osteoclastogenic medium or osteoblastic medium for indicated time. Then total RNA was isolated using RNeasy Mini Kit (Qiagen, Valencia, CA, United States) according to the instructions. cDNA was synthesized using Prime Script RT reagent kit (TaKaRa Biotechnology) and RT-PCR was performed using SYBR (TaKaRa Biotechnology). The GAPDH was used as the housekeeping gene to normalize the expression of target genes. All the primer sequences were as follows: GAPDH, F 5′-TGCACCACCAACTGCTTAG-3′ and R 5′-GGATGCAGGGATGATGTTC-3′; Cathepsin K, F 5′-AGGCATTGACTCTGAAGATGCT-3′ and R 5′-TCCCCA CAGGAATCTCTCTG-3′; TRAP, F 5′-CTGGAGTGCACGAT GCCAGCGACA-3′ and R 5′-TCCGTGCTCGGCGATGGACC AGA-3′; C-fos, F 5′-CCAGTCAAGAGCATCAGCAA-3′ and R 5′-AAGTAGTGCAGCCCGGAGTA-3′; NFATc1, F 5′-CCGT TGCTTCCAGAAAATAACA-3′ and R 5′-TGTGGGATGTG AACTCGGAA-3′; ALP, F 5′-CTTGACTGTGGTTACTGCTG ATCA -3′ and R 5′-GTATCCACCGAATGTGAAAACGT-3′; OC, F 5′-CTGACCTCACAGATCCCAAGC -3′ and R 5′-TG GTCTGATAGCTCGTCACAAG-3′; Runx2 F 5′-AGTCCCAA CTTCCTGTGCTCC-3′ and R 5′-CGGTAACCACAGTCCCA TCTG-3′; Osterix, F 5′-ACCAGGTCCAGGCAACAC-3′ and R 5′-GCAAAGTCAGATGGGTAAGTAG-3′.

### Western Blot Analysis

The isolated primary BMMs were seeded into 6 wells in the same density (2.5^∗^10^3^ cell/cm^2^) and cultured in α-MEM medium containing 15% FBS, 1% penicillin, and 10 ng/ml M-CSF for 24 h. After being pre-treated with DMSO or boldine (50 μM) for 2 h, the cells were stimulated with RANKL (5 ng/ml) for 0, 5, 15, 30, 60 min. For the rescue study, the isolated primary BMMs were also seeded into 6 wells in the same density, cultured for 24 h, then pre-treated with DMSO or boldine (50 μM) for 2 h. After that, the cells were stimulated with RANKL (5 ng/ml) in the presence of DMSO or SC79 (8 μg/ml) for 30 min. The total proteins from these cells were collected using radio immune precipitation assay (RIPA) lysis buffer (R0278, Sigma Aldrich). The BCA protein assay kit (23225, ThermoFisher) was used as the instructions to measure the concentrations of these proteins. Then ten micrograms proteins of each sample were loaded in 10% SDS-polyacrylamide gel and transferred to PVDF membranes (Millipore, MA, United States). After blocking in 5% non-fat milk for 1 h, the membranes were incubated with indicated primary antibodies at 4°C overnight. After two times washes, the membranes were incubated with corresponding secondary antibodies for 1 h. Then the immunoreactive proteins were detected by premium autoradiography films (E3012, Denville) using electrochemical luminescence reagent (ECL) (Millipore, MA, United States).

### Statistical Analysis

All the experiments were repeated at least three times. All the data were expressed as mean ± SD. The significant differences between different groups were calculated using SPSS 16.0 software (SPSS, Chicago, IL, United States) by one-way ANOVA with Tukey–Kramer honest significant difference (HSD) test. The unpaired *t*-test was used for comparisons between two groups. ^∗^*p* < 0.5, ^∗∗^*p* < 0.1, and ^∗∗∗^*p* < 0.001 were used to indicate significant differences.

## Author Contributions

KC, HLiu, C-hZ, W-tZ, and A-mC designed the experiments. KC, Z-tL, PC, W-tZ, SL, and QY performed the experiments. X-zJ, HLiu, Y-tW, and HLin performed the measurement and analysis. KC, V-JP, and HLiao drafted the manuscript. V-JP revised the manuscript. All authors have contributed to the final version and approved the publication of the final manuscript.

## Conflict of Interest Statement

The authors declare that the research was conducted in the absence of any commercial or financial relationships that could be construed as a potential conflict of interest.
